# Microstructural Analysis and Rheological Modeling of Asphalt Mixtures Containing Recycled Asphalt Materials

**DOI:** 10.3390/ma7096254

**Published:** 2014-09-02

**Authors:** Augusto Cannone Falchetto, Ki Hoon Moon, Michael P. Wistuba

**Affiliations:** 1 Pavement Engineering Centre (ISBS), Technische Universität Braunschweig, Beethovenstraße 51b, Braunschweig 38106, Germany; E-Mail: m.wistuba@tu-bs.de; 2 Material R&D Division, Samsung C&T Corporation, 5th Floor Daeryung Gangnam Tower, 826-20, Yeoksam 1-Dong, Gangnam-Gu, Seoul 135-935, Korea; E-Mail: moonx113@umn.edu

**Keywords:** recycling, reclaimed asphalt pavement, manufacturer waste scrap shingles, tear-off scrap shingles, microstructure, creep stiffness, back-calculation

## Abstract

The use of recycled materials in pavement construction has seen, over the years, a significant increase closely associated with substantial economic and environmental benefits. During the past decades, many transportation agencies have evaluated the effect of adding Reclaimed Asphalt Pavement (RAP), and, more recently, Recycled Asphalt Shingles (RAS) on the performance of asphalt pavement, while limits were proposed on the amount of recycled materials which can be used. In this paper, the effect of adding RAP and RAS on the microstructural and low temperature properties of asphalt mixtures is investigated using digital image processing (DIP) and modeling of rheological data obtained with the Bending Beam Rheometer (BBR). Detailed information on the internal microstructure of asphalt mixtures is acquired based on digital images of small beam specimens and numerical estimations of spatial correlation functions. It is found that RAP increases the autocorrelation length (ACL) of the spatial distribution of aggregates, asphalt mastic and air voids phases, while an opposite trend is observed when RAS is included. Analogical and semi empirical models are used to back-calculate binder creep stiffness from mixture experimental data. Differences between back-calculated results and experimental data suggest limited or partial blending between new and aged binder.

## 1. Introduction

Using recycled materials is a top priority in the pavement industry not only for reducing construction costs but also for minimizing the environmental impact of road construction. Over the past decades, the use of different recyclable materials has been investigated by international, national, and local agencies; the list includes Reclaimed Asphalt Pavement (RAP), reclaimed Portland cement concrete, iron blast-furnace slag, fly ash, waste tire rubber, waste glass, and roofing shingles. Two sources in particular—RAP and Recycled Asphalt Shingles (RAS)—have been increasingly used for asphalt pavement applications.

RAP has been used in the U.S. for more than three decades [1,2,3]. According to Federal Highway Administration (FHWA), almost 73 million tons of RAP are reclaimed and 84% of that reclaimed (62 million tons) is used annually in asphalt pavement construction, making RAP the most recycled material in the U.S. [4]. A number of specifications that regulate the amount of RAP have been already in place for many years. Most of these limits were developed from observations of field performance of asphalt pavements built with recycled mixture.

In Europe, the use of RAP is common practice with some countries having more than 30 years of experience, such in the Netherlands [5], Denmark and Germany [6]. More than 4000 production sites and over 10,000 companies are involved in RAP recycling and in the production and placing of asphalt materials. Roughly 50 million tons of RAP are produced in Europe each year with over 70% of it being reused for pavement surfaces [7].

Roofing shingles represent another material that is available in large quantity for recycling. According to one estimate, almost 11 million tons of waste bituminous roofing materials are generated each year in the U.S. and most of them are discarded into landfills [4,8,9]. The pavement recycling of roofing shingles finds limited application in Europe where this material is generally used for energy recovery as fuel supplement [10]. The use of RAS in hot mix asphalt has seen increased acceptance from government agencies and construction contractors in the U.S. only in recent years. The main obstacle is the potentially detrimental effect of the aged oxidized binder present in old roofing shingles.

## 2. Literature Review

A vast amount of literature in the past addressed the effect of RAP on recycled asphalt mixtures and, in most cases, it was concluded that RAP has a significant influence on materials’ properties [11,12,13,14,15]. Little *et al.* [11] and McDaniel *et al.* [16] observed that higher RAP content are associated with increased viscosity and stiffness at high and intermediate temperatures, positively affecting rutting resistance; nevertheless, limited relaxation properties and reduced fracture resistance were also experienced due to increased material brittleness. Other research efforts focused on design procedures, forensic evaluation and modeling [17,18]; specifications were also developed with recommendations for selecting RAP content based on traffic level. For example, the Minnesota Department of Transportation (MnDOT) Specification 2350/2360 [19] allows up to 40% (by weight) RAP based on traffic level and binder grade.

From a European perspective, two large research efforts were undertaken in the past under the umbrella of the European Union [14,15]; the main focus of these studies was on test methods and safe use of alternative pavement materials to obtain satisfactory environmental and functional performance. Two recent research projects [20,21] have addressed recycling through a holistic approach with the goal of raising the level of re-use of asphalt mixture with minimum material downgrading. The maximization of RAP recycling, together with the use of rejuvenator, was also addressed in the past [22] and in two ongoing research projects [23,24] in Germany, with the objectives of evaluating the field response of the material and to implement material selection recommendations for practitioners.

More recent studies centered their efforts in understanding and modeling the effective blending between virgin and aged binder in the recycled mixtures [25,26]. In particular, Kriz *et al.* [27] investigated the diffusion processes occurring at the interface between virgin and recycled binders concluding that the total thickness of the new-aged binder film plays a fundamental role in the process.

Regarding the re-use of roofing asphalt shingles, two distinct categories are available in the market: Manufacturer Waste Scrap Shingles (MWSS) and Tear-off Scrap Shingles (TOSS), from old roofs that have been exposed to solar radiation and high temperatures for extended periods of time. Both TOSS and MWSS, which are generally identified as RAS, contain a high amount of small aggregates and a much harder asphalt binder compared to that used in asphalt pavement mixtures: at 25 °C, the penetration values for asphalt binder in shingles ranges from 20 to 70 dmm, while traditional paving binders range from 50 to 300 dmm [28].

Newcomb *et al.* [28] were among the first to investigate the properties asphalt mixture prepared with different amounts of RAP, MWSS and TOSS. It was observed that adding MWSS did not significantly change the moisture susceptibility, however, TOSS did. Lower tensile strength was measured with increasing MWSS and TOSS contents. It was concluded that up to 5% of MWSS could be used in asphalt mixtures with a minimum impact on the mixture properties. However, addition of TOSS resulted in embrittled or stiffer mixture which is not desirable for cracking resistance at low temperatures. The 5% limit for MWSS was also proposed by other authors [29,30] that measured proportional decrease in tensile strength, creep stiffness and freeze-thaw resistance with increasing amount of roofing shingles. Burak and Ali [31] investigated several mechanical properties of asphalt mixtures containing roofing waste shingles from 1% to 5%. They observed that shingles could be used to improve Marshall-stability and rutting resistance. Other recent research efforts [32,33] investigated the cracking properties of mixture containing RAP and RAS, indicating that the use of rejuvenators and of a mix design which balances rutting and moisture damage, together with cracking requirements, can significantly improve the performance of the recycled mixture. McGraw *et al.* [34] evaluated the combined use of RAP and RAS showing the negative effect of TOSS on mixture strength and binder’s critical cracking temperature. In a recent study [35] on the use of fractionated recycled asphalt pavement (FRAP) and RAS, it was found that the fibers contained in RAS may be beneficial to low temperature cracking resistance. Recent European studies have also addressed the use of shingles [36,37]. In one of this research works [37], both stiffness and fatigue properties of the recycled mixture were investigated showing an increase in mixture stiffness due to the inclusion of RAP and shingles (up to 5%).

Reusing asphalt shingles in asphalt mixture poses, therefore, significant challenges especially in cold climates, where good fracture resistance is critical for good pavement performance. This is particularly true for TOSS, which contain highly oxidized binders that are more prone to brittle failure. Unlike MWSS, only recently a provisional specification (MnDOT tear-off scrap asphalt shingles specification 2360, 2010) on the use of TOSS was released which limits the content to 5% (by weight) with the provision that at least 70% (by weight) of the required binder is new binder [38].

## 3. Objective and Research Approach

The present study investigates the effect of adding different amounts of RAP, MWSS, and TOSS to asphalt mixtures used for pavement applications based on changes in asphalt mixtures microstructure, and on modeling of mixtures and binders’ low temperature properties, respectively.

The internal microstructure of asphalt mixture is analyzed using Digital Image Processing (DIP) of scanned Red Green Blue (RGB) scale images of small beam specimens having the same size of those used for creep tests in the Bending Beam Rheometer (BBR) [39]. Numerical evaluation of 2- and 3-point correlation functions, together with the estimation of the auto-correlation length (ACL), [40] of the aggregate, asphalt mastic, and air voids phases is performed to obtain detailed information on the microstructural spatial distribution.

The semi-empirical Hirsch [41] model and Huet [42] analogical model [43] coupled with transformation proposed by the research team of the Ecole Nationale des Travaux Publics de l’Etat (ENTPE) [44,45], are used to investigate the effect of adding recycled material on both asphalt binder and asphalt mixture creep stiffness. Back-calculation of the asphalt binder creep stiffness is performed using mixture creep stiffness data obtained with the Bending Beam Rheometer (BBR) [39,46].

The objective is to determine if changes in mixture behavior are due to variations in microstructure, from addition of recycled material, or to the effective blending of new and aged binder, or to a combination of both phenomena.

## 4. Materials and Testing

Eight different asphalt mixtures (Table 1) provided from the Minnesota Department of Transportation (MnDOT) to the Department of Civil Engineering at University of Minnesota were used in this study. The entire set of mixtures was prepared with a virgin plain binder having Performance Grade (PG) PG58-28 [47] and target air voids content of 4%. Three types of virgin aggregates were blended to prepare the mixtures: pit-run-sand, quarried ¾ inch (19 mm) dolostone, and quarried dolostone manufactured sand. The recycled material consisted of different amounts of RAP, TOSS and MWSS: Table 2 presents the gradation of each recycled material used. Additional details on these materials can be found elsewhere [48].

**Table 1:** Mix design for the eight asphalt mixtures investigated.

Mix		Recycled Material			*VMA* (%)	*VFA* (%)	*V_Air Voids_* (%)	*V_Mastic_* (%)
ID	Description	RAP (% weight)	TOSS (% weight)	MWSS (% weight)				
1	PG 58-28 Control	0	0	0	15.9	76.6	3.7	12.2
2	15% RAP	15	0	0	15.2	72.9	4.1	11.1
3	25% RAP	25	0	0	15.3	73.0	4.1	11.2
4	30% RAP	30	0	0	15.0	75.4	3.7	11.3
5	15% RAP 5% MWSS	15	0	5	15.6	75.0	3.9	11.7
6	15% RAP 5% TOSS	15	5	0	15.9	77.2	3.6	12.3
7	25% RAP 5% TOSS	25	5	0	15.4	73.9	4.0	11.4
8	25% RAP 5% MWSS	25	0	5	14.8	72.5	4.1	10.7

**Table 2:** Gradation of RAP MWSS and TOSS.

Sieve size (mm)	RAP Passing (%)	MWSS Passing (%)	TOSS Passing (%)
19.000	100.0	100.0	100.0
12.500	94.0	100.0	100.0
9.500	87.0	100.0	100.0
4.750	69.0	100.0	98.0
2.360	55.0	99.0	97.0
1.180	44.0	85.0	81.0
0.600	32.0	65.0	61.0
0.300	18.0	49.0	53.0
0.150	10.0	35.0	40.0
0.075	6.6	24.1	30.9

**Figure 1:**
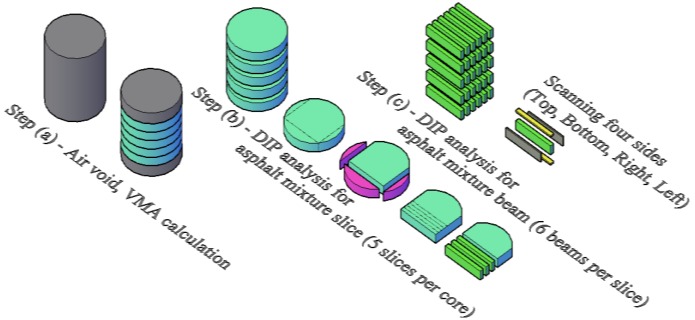
Specimens preparation for (digital image processing) DIP analysis and Bending Beam Rheometer (BBR) testing.

The eight asphalt mixtures were compacted into cylindrical specimens with a Superpave gyratory compactor (SGC). Five slices of asphalt mixture and, from these, specimens having the same size of BBR beams were cut and used for DIP analysis, first, and for BBR creep tests at low temperature in a second stage. Specimens were prepared and tested according to the procedure proposed by Marasteanu *et al.* [46]; a scheme of the different preparation steps is shown in Figure 1.

**Figure 2:**
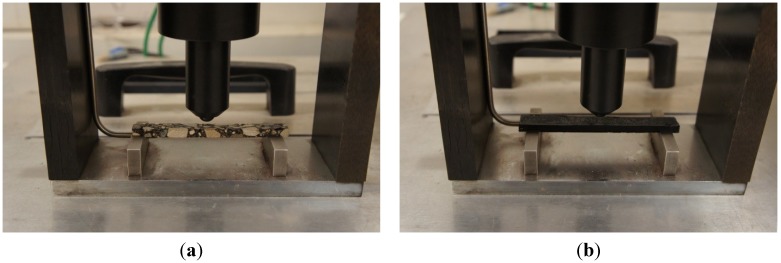
Bending Beam Rheometer with thin (**a**) asphalt mixture and (**b**) asphalt binder beams.

Asphalt mixtures beams (six replicates per mixture obtained from the central slice) were tested using the Bending Beam Rheometer (Figure 2a), and creep stiffness, *S*(*t*), was then calculated as:

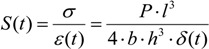
(1)
where *σ* is maximum bending stress in the beam; *ε*(*t*) is the bending strain; *P* is the constant load applied at the mid-span of the specimen; *l* is the beam length (span) (102.0 ± 5 mm); *b* is the width of the specimen (12.7 ± 0.5 mm); *h* is the height of the specimen (6.25 ± 0.5 mm); *δ*(*t*) is the time dependent deflection at the mid-span of the beam; and *t* is the time variable. Due to the higher stiffness of asphalt mixtures, the load was increased to 4000 mN and test duration was set to 1000 s.

BBR tests [39] were also performed on the asphalt binders extracted from mixtures 2, 3, 4, 5, 6, 7 and 8 and, then, used for comparison purposes with the models’ predictions; extraction and asphalt binder tests were conducted at MnDOT Office of Materials (Figure 2b). In addition, BBR creep stiffness was measured on the original PG58-28 binder after short term aging with the Rolling Thin-Film Oven Test (RTFOT) [49]; this value was assumed as control in the analysis. All binder tests were performed according to AASHTO T313-12-UL [39] and, therefore, limited to 240 s under a constant load of 1000 mN. To reduce errors associated with time-temperature superposition, binder and mixture creep stiffness were obtained at the same test temperature of −6 °C, which is equal to the binder PG low temperature limit +22 °C.

## 5. Microstructural Analysis

Most research efforts have addressed asphalt mixture either as a two-phase material [50,51], or as a three-phase material [52,53]. Eriksen and Wegan [54] and Yue *et al.* [55] were among the first to investigate the microstructure of asphalt binders and mixtures, using DIP techniques, for obtaining material properties such as gradation, aggregate distribution, shape and orientation. Dai *et al.* [56] used DIP analysis to provide detailed information of asphalt mixture for Finite Element simulations of Indirect Tensile (IDT) strength test [57]. Kose *et al.* [58] and Partl *et al.* [59] and, more recently, other authors [60,61,62] have proposed advanced DIP techniques, and threshold algorithms for aggregate, asphalt mastic and air voids segmentation using RGB (Red-Green-Blue) scanned and gray scale converted asphalt mixture images. In some of these studies, the analysis of the materials microstructure was extended to a three-dimensional investigation based on 3D X-Ray Computer Tomography (CT) technology.

The microstructural information obtained with DIP techniques can be also used together with high-order microstructure functions, such as *n*-point correlation functions, to obtain improved upper and lower bounds of mechanical material properties [63]. Effective mechanical properties and failure characteristics of a heterogeneous material with the same volume fraction can be significantly influenced by the spatial distribution of its constituents [64]. In a recent study, Velasquez *et al.* [65] used DIP analysis and 2- and 3-point correlation functions to obtain specific input parameters for micromechanical models and detailed information on the Representative Volume Element (RVE) of asphalt mixture.

### 5.1. Two- and Three-Point Correlations: Theoretical Background

Correlation functions quantify the degree of influence among different parts of a material providing a numerical estimation of how they affect each other [62]. In the case of random heterogeneous materials, higher-order microstructural information is critical for describing the mechanical properties; several functions such as the *n*-point correlation functions, surface correlation functions, and lineal path functions, can be used for this purpose [63,65,66,67,68]. Among these set of functions, 2*-* and 3*-*point correlation functions present a simpler formulation compared to other complex statistical tools.

The *n*-point spatial correlation function measures the probability of finding all *n* points located on the space occupied by one of the phases of a heterogeneous material [40]. For instance, the 1-point correlation function, *S_1_*, measures the probability that any point lies on phase 1 and this corresponds to the volumetric fraction of phase 1. The 2-point correlation function, *S_2_*, calculates the probability that two points, separated by a certain distance, *r*, are located both in the same phase. Similarly, the 3-point correlation function, *S_3_*, computes the probability that all the vertices of a triangle are located in the same phase.

The *n*-point correlation function of a two-phase random heterogeneous material in a *d*-dimensional Euclidian space, *R^d^*, is defined according to Torquato [63] as:

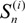
(*x*_1_,*x*_2_,*x*_3_…,*x*_n_) = ⟨*I*^(*i*)^(*x*_1_)*I*^(*i*)^(*x*_2_)*I*^(*i*)^(*x*_3_)*I*^(*i*)^(*x*_n_)⟩
(2)
where ⟨ ⟩ indicates the ensemble averaging. The indicator function *I*^(*i*)^(*x*) is defined as:

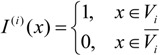
(3)
where *V_i_* ϵ *R^d^* is the volume occupied by the *i^th^* phase and *V_i_* ϵ *R^d^* is the volume occupied by the other phase.

The *n*-point correlation function is translationally invariant for a statistically homogeneous material. The function depends on the differences in the coordinate values of the *x_i_* vectors, but not on their absolute position [63]. The *n*-point correlation function can also be expressed as:

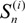
(*x*_1_,*x*_2_,*x*_3_…,*x*_n_) = 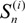
(*x*_12_,*x*_13_,*x*_14_…,*x*_1n_) for *n*≥1
(4)
where *x_ij_* = *x_j_* − *x_i_* is the difference between the two vectors *x_i_* and *x_j_*, and *x_1_* is the reference vector.

The 1-point correlation function represents the volume fraction *ϕ_i_* of the selected *i^th^* phase; this is constant and it denotes the probability that a randomly selected point in the material belongs to specific phase:

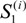
 = ⟨*I*^(*i*)^(*x*)⟩ = *ϕ_i_*(5)


The 2- and 3-point correlation function can be defined, respectively as [63]:

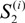
(*x*_1_,*x*_2_) = ⟨*I*^(*i*)^(*x*_1_)*I*^(*i*)^(*x*_2_)⟩
(6)

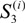
(*x*_1_,*x*_2_,*x*_3_) = ⟨*I*^(*i*)^(*x*_1_)*I*^(*i*)^(*x*_2_)*I*^(*i*)^(*x*_3_)⟩
(7)


In the case of a translationally invariant isotropic material, the 3-point correlation function can be expressed as:

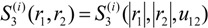
(8)
where *r_1_* = *x_2_* − *x_1_* and *r_2_* = *x_3_* − *x_1_* are vectors, and *u_12_* is the cosine of the angle *θ_12_* between vectors *r_1_* and *r_2_*:

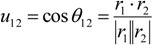
(9)


In addition, if the microstructure of the material does not present long range order, the initial value of 2- and 3-point correlation functions is *ϕ_i_* (*r* = 0) and for very large *r* (*r*→∞), it reaches the asymptotic limits of *ϕ_i_*^2^ and *ϕ_i_*^3^, respectively.

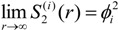
(10)

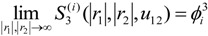
(11)


The *n*-point correlation function can be used to describe the microstructure of random heterogeneous materials such as asphalt mixtures; however, the computation process is complicated when an analytical solution is sought. Therefore, alternative techniques are required: a combination of DIP analysis and numerical algorithms provides a simpler approach for obtaining the values of the function [62,63,65].

### 5.2. Digital Image Processing (DIP) Analysis Overview

The internal structure of random heterogeneous materials, such as asphalt mixtures, can be described and analyzed using DIP techniques. A digitally converted image can be considered as a two dimensional independent function, *f*(*x*,*y*), where *f* is the function of color intensity and *f*(*x*,*y*) returns the color intensity of the image at the specific point (*x*,*y*). The entire digital image is made of the contribution of each pixel, which is associated to a specific location and intensity value [69]. For example, the matrix representation of an *R*×*C* size binary image (*i.e.*, consisted of black (0) and white (1) colors) is expressed as:

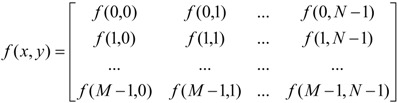
(12)


In this study, a flat-bed scanner was used to acquire 2D images of asphalt mixture in RGB (Red, Green and Blue) color format. Scanning resolution was limited to 720 dpi since this is sufficient to detect aggregate particles larger than 75 μm while reducing the time-consuming process of analyzing very large files. In previous studies [56,65], RGB scale asphalt mixture images were converted into binary images (black color for air voids + asphalt binder + aggregates smaller than 75 μm, and white color for aggregates larger than 75 μm) using noise filtering function. In the present research, RGB scale images of asphalt mixture were converted, through the MATLAB™ Image Processing Toolbox [70], into 8-bit gray scale images (intensity ranges from 0, black, to 255, white) to retain more information of the material phases.

The following steps schematically summarize the DIP analysis procedure used in this research effort; detailed information about the DIP analysis technique used in this study can be found elsewhere [62].

1. First, the volume fraction of the actual air voids content and of the Voids in the Mineral Aggregate (*VMA*, %) were obtained for each mixture using the experimental data as:

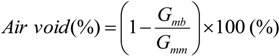
(13)

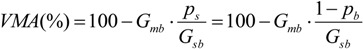
(14)

where: *G_mb_* is the bulk specific gravity; *G_mm_* is the maximum specific gravity; *p_s_* is the aggregate content (mass); *p_b_* is the asphalt content (mass); and *G_sb_* is the bulk specific gravity of aggregate. Then, from Equation (14), *VMA*, which includes air voids (*V_air_*) and the effective volume fraction of asphalt binder (*V_be_*), was expressed as:

*VMA* = *V_air_* + *V_be_* (15)

Based on the scanning resolution (*i.e.*, 720 dpi), the following expression was used for computing *VMA* [62]:

*VMA* = *V_air_* + (*V_be_* + *V_agg<75μm_*) = *V_air_* + *V_mastic_* (16)

Equations (13), (14) and (16) were next used to determine the volume fraction of mastic, *V_mastic_*.

2. Five slices, with thickness of 12.7 ± 5.0 mm, were cut from each asphalt mixture core, (Figure 1, Steps a and b) and then scanned in RGB scale. The digital images of the asphalt mixture slices (3540 pixels × 3540 pixels) were then converted to gray scale and noise filtering applied [62,70].
3. Based on the experimental data of Table 1, two threshold values, one for the air voids volume fraction, T1, and one for the mastic volume fraction, T2, were calculated from the converted gray scale images of asphalt mixture slices. The values of T1 and T2 were computed by assuming that the air voids phase takes the darkest pixel range in the gray scale images (Figure 1, Step b).
4. All the asphalt mixture slices were cut into 240 asphalt mixture BBR beams (8 mixtures × 5 slices × 6 replicates) [46] (Figure 1, Step b). The four largest sides of the BBR mixture beams were then scanned and converted into gray scale images [62]. Therefore, a total of 960 images of asphalt mixture beams were used for DIP analysis (Figure Step c). Finally, based on the threshold values, T1 and T2, previously obtained, three-phase images of asphalt mixture beams were generated; meanwhile the DIP values of VMAd (total VMA,%), Vagg_d (volume fraction of aggregate,%), Vma_d (volume fraction of asphalt mastic,%) and Vair_d (volume fraction of air voids,%) were computed.

**Figure 3:**
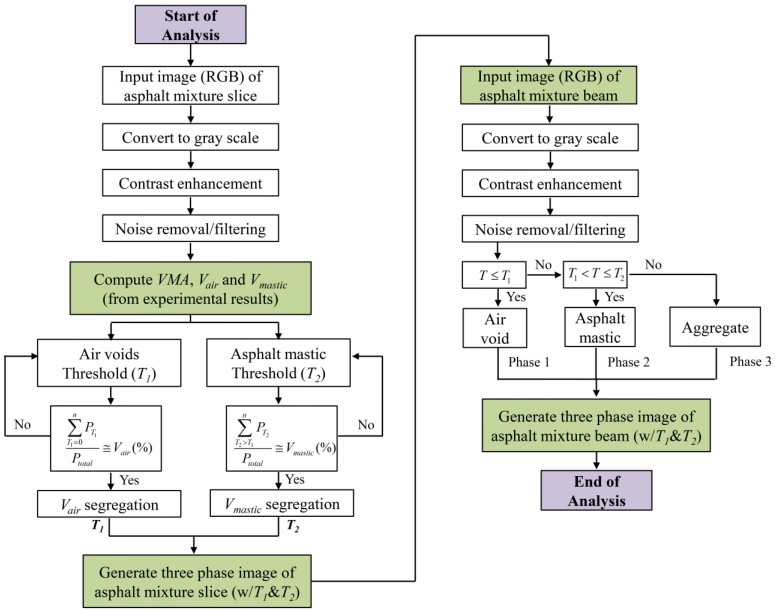
Flowchart of DIP analysis for generating three-phase images of asphalt mixtures. Notes: *P*: Number of pixels in the image; *T*: Threshold values (0:Black~255:White). In computing *T_1_* and *T_2_*, error level was converged to 5% [0 < *T_1_* < *T_2_* < 255].

The flowchart of the entire DIP analysis procedure used in the present study for generating three-phase (aggregate, asphalt mastic and air voids) images of asphalt mixture is shown in Figure 3.

**Figure 4:**
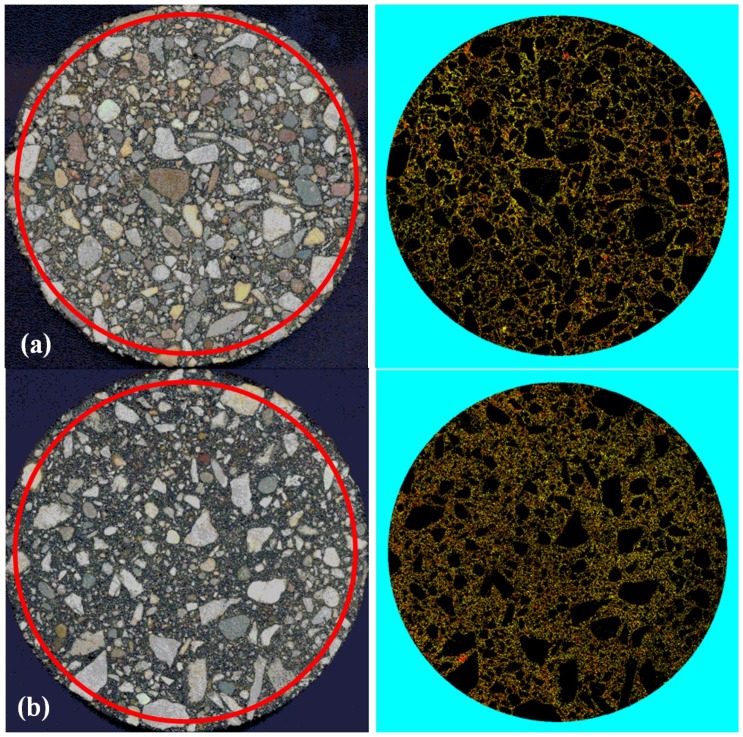
Images of asphalt mixture slices: original RGB and three-phase processed image. (**a**) Mixture 1; (**b**) Mixture 5.

**Figure 5:**
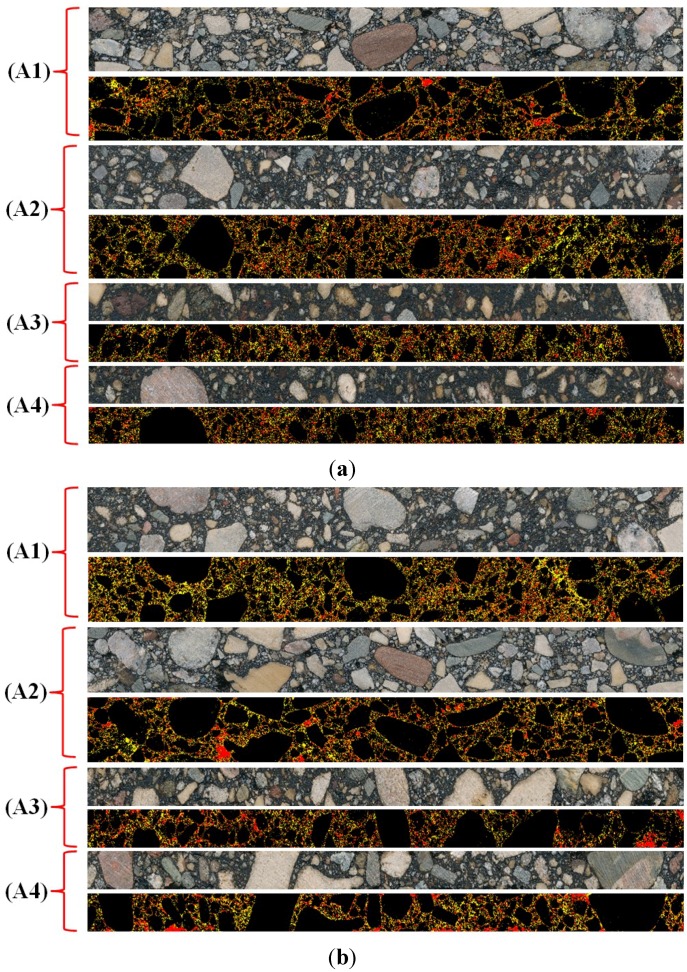
Original RGB and three-phase images of asphalt mixture beams: (A1) top, (A2) bottom (A3) right and (A4) left sides. (**a**) Mixture 1; (**b**) Mixture 5.

Table 3 presents the average values of *VMA _d_*, *V_ma_d_* and *V_air_d_*, obtained from DIP analysis. Figure 4 and Figure 5 provide examples of three-phase images obtained through DIP analysis, for two asphalt mixture slices and for two beams made of two different mixture types.

**Table 3:** Summary of the volumetric properties of asphalt mixture beams obtained from DIP analysis.

Mixture	*VMA_d_*, %		*V_air_d_*, %		*V_mastic_d_*, %		VMA*	V_air_*	V_mastic_*
	Ave	CoV	Ave	CoV	Ave	CoV	%	%	%
1	15.55	3.26	4.25	10.12	11.30	7.32	−0.35	0.55	−0.88
2	14.56	3.62	4.36	4.19	10.20	4.18	−0.64	0.26	−0.88
3	14.94	5.03	4.71	6.13	10.22	6.94	−0.36	0.61	−0.94
4	14.77	4.73	4.31	4.39	10.46	6.06	−0.23	0.61	3.65
5	14.25	6.77	4.31	5.87	9.94	10.42	−1.35	0.41	−1.76
6	15.08	3.70	4.11	4.46	10.97	5.55	−0.82	0.51	−1.31
7	14.42	3.44	4.64	4.17	9.79	5.77	−0.98	0.64	−1.59
8	14.08	2.91	4.67	6.53	9.40	6.46	−0.72	0.57	−1.33

Notes: VMA* = (VMA − VMA_d_), V_air_* = (V_air_ − V_air d_), V_mastic_* = (V_mastic_ − V_mastic d_); Values of V_air_, and V_mastic_, and VMA can be referred from Table 1.

Figure 4 and Figure 5 suggest good correspondence of the material phases between the original RGB-scale images and the three-phase image of asphalt mixture, indicating that the experimental volumetric parameters can be successfully used as input for DIP analysis. In addition, low values of Coefficient of Variation (CoV_max_ = 10.42%) were observed in all analyzed mixtures, including those containing RAP and RAS together (Table 2). This indicates that there is a consistently similar volume fractions distribution of the material phases among the different asphalt mixture beams cut from the same asphalt mixture cylinder.

### 5.3. Numerical Solutions of 2- and 3-Point Correlation Functions

The 2-point correlation function can be obtained using a discretized expression of Equation (6) accounting for the width and the height of the specific digital image. Nevertheless, when high resolution images containing a large number of pixels are used, a “brute force method” may be very time demanding and sometimes even prohibitive. Recently, an algorithm, based on Monte Carlo simulation and on binary images of the asphalt mixture BBR specimens, was proposed for calculating the correlation functions curves [65]; a simplified version of this method, applied to three-phase images, is used hereafter to determine the values of 2- and 3-point correlation functions of each asphalt mixture phase [62].

**Figure 6:**
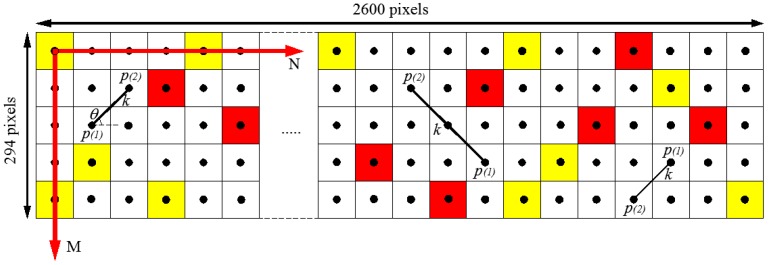
Schematic of the simplified algorithm for 2-point correlation function calculation.

The procedure for computing 2-point correlation consists in dropping vectors of increasing length and random orientation (*i.e.*, angle *θ*) in the digital image *N* times, while the number of times that both end points of the vectors are in the phase of interest is recorded (Figure 6). The process is iterated for vectors of lengths varying from 0 to half the size of the image for a number of drops *N* > 100,000 [62,65].

**Figure 7:**
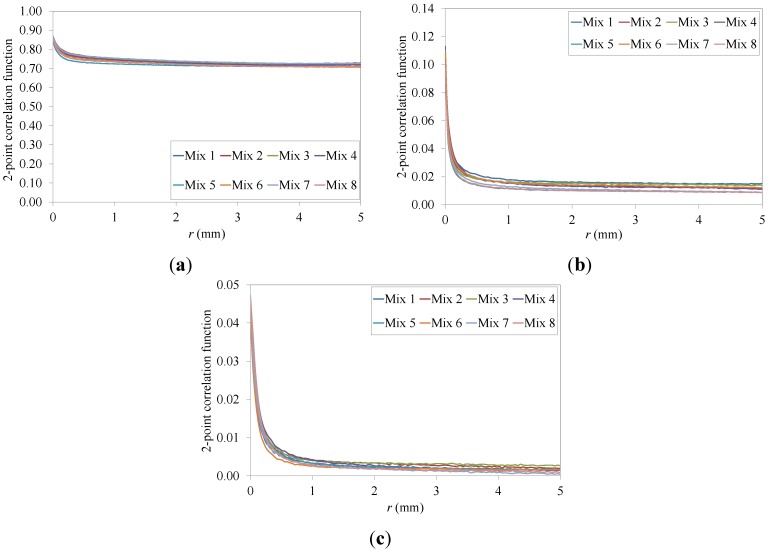
Two-point correlation functions for the entire set of eight asphalt mixtures used: (**a**) Aggregates; (**b**) Asphalt mastic; (**c**) Air voids.

The three-phase images of the different beam specimens were used to compute 2-point correlation functions of aggregates, asphalt mastic and air voids. The single values of the 2-point correlation function of each specimen were calculated as the mean of the two larger sides of each beam image. Figure 7 presents the curves of the average 2-point correlation function for all the eight mixtures investigated. Small CoV were obtained: for example, in the case of the aggregate phase, a CoV_max_ = 3.8% was observed.

The values of the 2-point correlation function do not fluctuate as the distance (*r*) increases for all eight mixtures investigated. The 2-point correlation function starts at approximately *ϕ_i_* and smoothly drops to 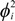, corresponding to the asymptotic limit of Equation (10) as *r* approaches infinite [40,63]. All the curves are very close although not overlapping, suggesting a slightly different spatial arrangement of the three phases across the different mixtures most likely associated to the different amount of recycled material used in the mix design.

**Figure 8:**
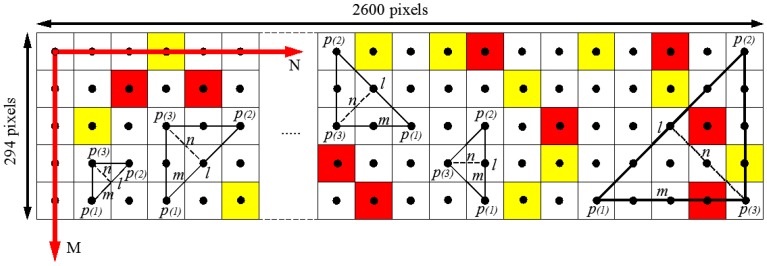
Schematic of the simplified algorithm for 3-point correlation function calculation.

For the calculation of the 3-point correlation function, a set of lattice commensurate triangles [65] and the symmetry property of the function can be used to reduce the computational time. The set of triangles used for the estimation of the spatial correlation function are defined by three integers, *l*, *m*, and *n* (Figure 8) which are related by the following conditions:

*m* ≤ *l*/2 and *m*^2^ + *n*^2^ ≤ 2*ml* (17)

Each triangle defined by the integers (*l*, *m*, *n*) is randomly dropped *N* = 100,000 times in the three-phase image [62]. The number of times (*N_hits_*) the three vertices of the triangle are in the phase of interest is counted (Figure 8).

**Figure 9:**
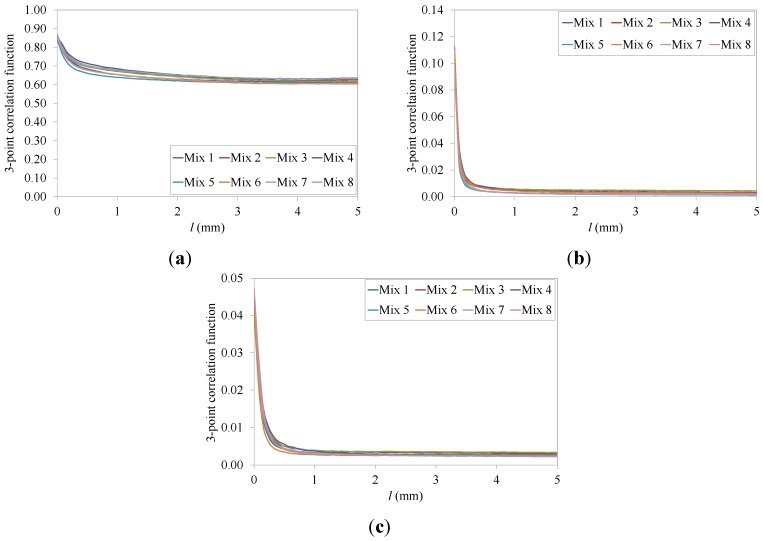
Three-point correlation functions for the entire set of eight asphalt mixtures used: (**a**) Aggregates; (**b**) Asphalt mastic; (**c**) Air voids.

In addition, to further reduce computation time and memory demand, only symmetrical triangles in the size range of 0.0 mm < *l* ≤ 5.2 mm were used. Although this restriction does not allow obtaining the all of the 3-point correlation functions, this method significantly reduces the computation time (from 4~5 h to 2~3 min for each simulation) while still providing important information on the global trend of the function [62]. The curves of the average 3-point correlation function for all eight mixtures investigated are shown in Figure 9.

Similarly to the 2-point correlation function, small CoV were observed with a CoV_max_ = 4.1% for mixture 1. The average 3-point correlation function has a similar pattern for all eight asphalt mixtures considered. No large fluctuations in the spatial distribution of the materials phases were observed as the size of the triangle increases. *S_3_* begins at *ϕ_i_* and smoothly drops to 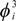, as described by Equation (11), showing no evidence of clustering or preferred paths in the microstructure [62,63]. Also, in this case, the curves of the function are close, but not overlapping, confirming the influence of the recycled materials on the distance required for the function to reach a stable value.

### 5.4. Autocorrelation Length

Based on the results of 2- and 3-point correlation functions, it was decided to investigate the auto correlation length (ACL) associated with the correlation function of each mixture. ACL measures how quickly a random event decays, or the distance over which two or three points can be treated as independent for the specific random process [40,62,71]. For example, Bazant and Pang [72] associated the ACL with the estimation of the dimensions of the Representative Volume Element (RVE) of the material. Some authors [40,71,73] used exponential expressions to analytically describe the autocorrelation functions (ACF) and identified the ACL as the distance over which the ACF decays to 1/*e* of its original value [73]; however, it was recently shown that this may not apply to asphalt mixture [62]. Therefore, in this study, ACL was estimated, for all the material phases, as the distance over which the function assumes its theoretical value (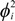 and 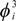 for 2- and 3-point correlation functions, respectively) in a stable manner within a 5% error (95% confidence interval). The following ACL values were obtained (Table 4).

**Table 4:** Auto Correlation Length (ACL) of 2- and 3-point correlation functions.

ID	Material	2-Point correlation function (mm)			3-Point correlation function (mm)		
		Aggregates	Mastic	Air voids	Aggregates	Mastic	Air voids
1	PG 58-28 Control	3.97	0.59	1.02	2.33	0.40	0.88
2	15% RAP	3.97	0.64	1.48	2.34	0.48	1.37
3	25% RAP	4.06	0.64	1.44	2.42	0.48	1.53
4	30% RAP	4.09	0.66	1.51	2.45	0.49	1.61
5	15% RAP 5% MWSS	1.57	0.25	0.72	1.37	0.22	0.73
6	15% RAP 5% TOSS	2.75	0.42	0.76	2.10	0.32	0.78
7	25% RAP 5% TOSS	2.84	0.47	1.14	2.18	0.40	0.97
8	25% RAP 5% MWSS	2.03	0.30	0.76	1.69	0.28	0.76

From Table 4, it can be first observed that the values of ACL increase with respect to the three phases: aggregates, air voids and asphalt mastic, respectively. This indicates that ACL reflects the influence of the spatial distribution of each material phase rather than the overall volume fraction. The addition of RAP results in larger ACLs for all phases, suggesting a longer randomness range and thus larger RVE dimensions.

A quite significant opposite trend is associated with the presence of RAS. It may be hypothesized that this is due to the higher amount of fine particles which are present in shingles. Nevertheless, the aged TOSS material seems to introduce a smaller randomness decay compared to the MWSS. It is interesting to note that the ACL of the 2-point correlation function is larger than that observed for the 3-point correlation function, suggesting a slightly faster decay of the microstructure randomness associated to the potential presence of clustering effect and/or connected paths. In addition, the maximum value of ACL was found to be equal to 4.09 mm (Mixture 4, Aggregate phase) which is smaller than the thickness (=6.35 mm) and width (=12.70 mm) of the BBR beam used for mixture testing. Therefore, BBR specimens can be assumed to be large enough for obtaining the low temperature creep properties of asphalt mixture.

## 6. Rheological Analysis

Based on the results of the microstructural analysis of the eight asphalt mixtures evaluated in the present study, the change in rheological properties due to the addition of aged recycled materials is investigated in the next section.

### 6.1. Analogical and Semi Empirical Models

**Figure 10:**
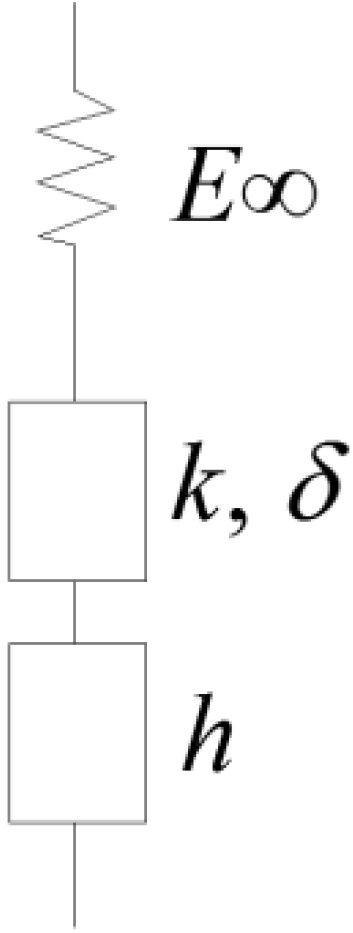
Huet model [42].

In previous research efforts [26,74], analogical models were used to obtain the creep stiffness of asphalt binders from the creep stiffness of the corresponding asphalt mixtures (inverse problem). It was found that Huet model [42] fitted very well the experimental data obtained from BBR tests at low temperatures for both asphalt binders and mixtures. This model is composed of two parabolic elements, *D_1_*(*t*) = *a_1_*(*t*/*τ*)*^h^* and *D_2_*(*t*) = *a_2_*(*t*/*τ*)*^k^*, and a spring with stiffness *E*_∞_, combined in series (Figure 10).

The analytical expression of the Huet model for creep compliance is:

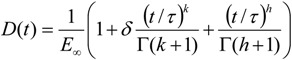
(18)
where *D*(*t*) is the creep compliance; *E*_∞_ is the glassy modulus; *h*, *k* are exponents such that 0 < *k* < *h* < 1; *δ* is a dimensionless constant; *t* is the time, *Γ* is the gamma function, *τ* is the characteristic time varying with temperature accounting for the Time Temperature Superposition Principle (TTSP): *τ* = *a_T_*(*T*)*τ*_0_(*T_S_*); *a_T_* is the shift factor at temperature *T*; *τ_0_* is the characteristic time determined at reference temperature *T_S_*.

The authors [26,74] found the following relationship between the characteristic time of mixture, *τ_mix_* and the characteristic time of the corresponding binder, *τ_binder_*, at the reference temperature *T*:

*τ_mix_*(*T*) = 10^*α*^*τ_binder_*(*T*) (19)

where *α* is a regression coefficient depending on mixture type and aging [45]. This expression corresponds to the Equation proposed by Di Benedetto [45] and obtained from 2S2P1D model and complex modulus data [44,45]. Based on Equations (18) and (19), a transformation that relates the creep stiffness of the asphalt binder *S_binder_*(*t*) to the creep stiffness of the corresponding asphalt mixture *S_mix_*(*t*) can be written:

*S_mix_*(*t*) = *S_binder_*(*t*10^*−α*^) ∙ (*E_∞_mix_*/*E_∞_binder_*) (20)

where *E_∞_mix_* is the mixture glassy modulus and *E_∞_binder_* is the binder glassy modulus. Expression (19) does not depend on the original model used to obtain it and represents the low temperatures formulation of the ENTPE transformation [44,45,74].

A different back-calculation method based on the semi empirical Hirsch [41] formulation was used by several authors [17,65,74,75]. In this model, the effective response of the material is obtained assembling the mixture components (air voids, asphalt binder and aggregate) in parallel and in series. The general expression is:

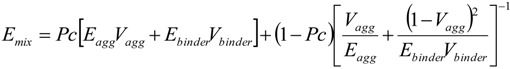
(21)
where *E_mix_* is the effective modulus of the mixture, *E_agg_* and *V_agg_* are the modulus and volume fraction of the aggregate; *E_binder_* and *V_binder_* are the modulus and volume fraction of binder; *P_c_* = 0.1ln(*E_binder_*/*a*) + 0.609 is the contact volume (an empirical factor); *E_binder_* is the relaxation modulus of the binder in GPa; and *a* is a constant equal to 1 GPa.

### 6.2. Back-Calculation

In this section, asphalt binder creep stiffness is back-calculated from the creep stiffness experimental data of asphalt mixture with the aim of obtaining useful information on the blending process and the degree of blending between the new virgin binder and the aged binder contained in RAP and RAS. For such a purpose, Huet model was introduced into Equation (11) as:

*S_mix_*(*t, h, k, δ, τ_mix_*) = *S_binder_*(*t, h, k, δ, 10^*α*^τ_mix_*) ∙ (*E_∞_mix_*/*E_∞_binder_*) (22)

where the five constants *δ*, *k*, *h*, *E_∞_*, and *τ* can be estimated by minimizing the sum of the distances between asphalt mixture experimental data and the predicted values obtained from Equation (22) at *n* time points. In previous studies [44,45,74], it was found that Huet model parameters assume the same values for binder and corresponding mixtures. Therefore, the difference between asphalt binder and asphalt mixture is given by the characteristic times, *τ_binder_* and *τ_mix_*, which, based on Equation (19), are directly associated to parameter α. In this study, the value of α was obtained during the minimization process starting from initial values of *δ*, *k*, *h*, *E_∞_*, *τ* found in literature [42,44,45,74]. In addition, the recent recommendation proposed by Moon *et al.* [76], which limits the value of *k* between 0.25 and 0.40 for plain binder having PG58-28, was used. Table 5 presents the Huet model parameters for the eight asphalt mixtures tested at −6 °C and for the corresponding back-calculated asphalt binders; the values of α are given in the last column of the table.

**Table 5:** Huet model parameters for mixtures and corresponding back-calculated binders.

ID	Material	*δ*	*k*	*h*	*E*_∞_binder_ (MPa)	*E*_∞_mix_ (MPa)	Log (*τ*_binder_)	Log (*τ*_mix_)	*α*
1	PG 58-28 Control	6.67	0.28	0.71	2979	29,895	−0.770	2.390	3.16
2	15% RAP	6.45	0.25	0.58	2982	29,921	−0.824	3.646	4.47
3	25% RAP	6.02	0.30	0.57	2985	29,926	−0.658	3.492	4.15
4	30% RAP	6.83	0.25	0.62	2980	29,899	−0.824	3.726	4.55
5	15% RAP 5% MWSS	6.84	0.27	0.64	2988	29,931	−0.509	3.591	4.10
6	15% RAP 5% TOSS	6.85	0.25	0.66	2986	29,932	−0.456	4.274	4.73
7	25% RAP 5% TOSS	6.04	0.28	0.59	2989	29,926	−0.409	4.171	4.58
8	25% RAP 5% MWSS	6.11	0.28	0.59	2991	29,962	−0.495	3.965	4.46

Table 5 presents values of parameters *h* and *k* which are in agreement with the values obtained in different research efforts [45,74,76], while *δ* is higher compared to what was found in literature. As expected, there is a significant difference in the characteristic times of binders and corresponding mixtures. Nevertheless, it is interesting to observe that the presence of recycled materials significantly influences this parameter [25], although there is not a clear common trend for binder and mixture. Higher values of the transformation parameter, α, were observed when recycled material is added to the mixture in comparison with the control mixture prepared with virgin aggregates and virgin binder. This is true both when only RAP is used as well as when MWSS and TOSS are included in the mix design. Nevertheless, no statistically significant differences could be detected among the values of α obtained for the recycled mixtures. Furthermore, no specific trend of the parameter *α* could be identified and associated with the amount of recycled material.

**Figure 11:**
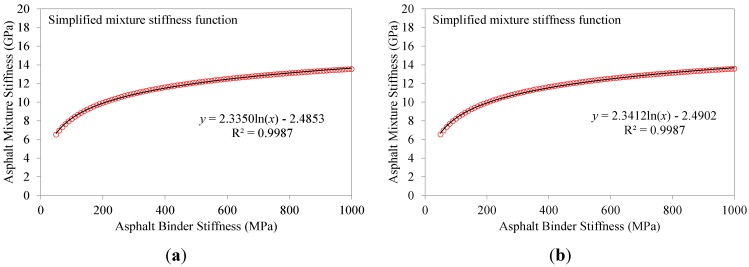
Simplified mixture stiffness function for mixtures 1 (**a**) and 5 (**b**), T = −6 °C.

In addition to the ENTPE transformation, back-calculation of asphalt binder creep stiffness was performed with the Hirsch model for comparison purposes. The simplified procedure adopted includes three steps. First, based on the volumetric properties of the mixtures (Table 1) and on Equation (21), plots of binder creep stiffness *versus* predicted mixture stiffness were generated for binder stiffness values between 50 and 1000 MPa. Figure 11 shows the predicted curves for mixtures 1 and 5.

Then, the following expression, *E_mix_* = *a*Hln(*E_binder_*) + *b*, was fitted to log of mixture stiffness *vs.* log of binder stiffness curves and parameters *a* and *b* were determined. Finally, the binder stiffness was simply calculated over the entire range of loading times.

### 6.3. Comparison with Experimental Data

**Figure 12:**
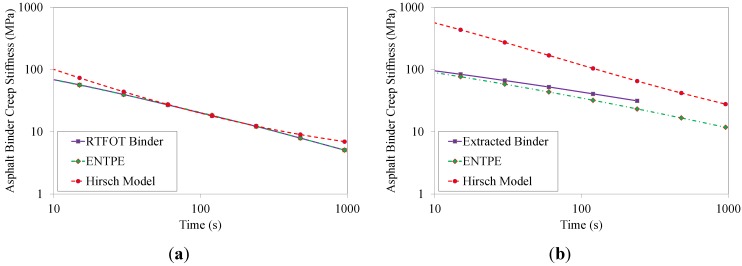
Back-calculated and extracted creep stiffness of asphalt binder: mix 1 (**a**) and 2 (**b**), T = −6 °C.

**Figure 13:**
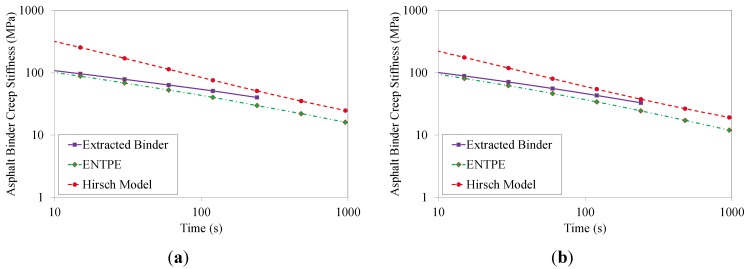
Back-calculated and extracted creep stiffness of asphalt binder: mix 3 (**a**) and 4 (**b**), T = −6 °C.

**Figure 14:**
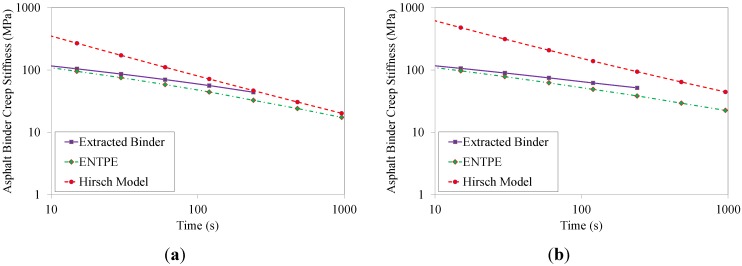
Back-calculated and extracted creep stiffness of asphalt binder: mix 5 (**a**) and 6 (**b**) , T = −6 °C.

**Figure 15:**
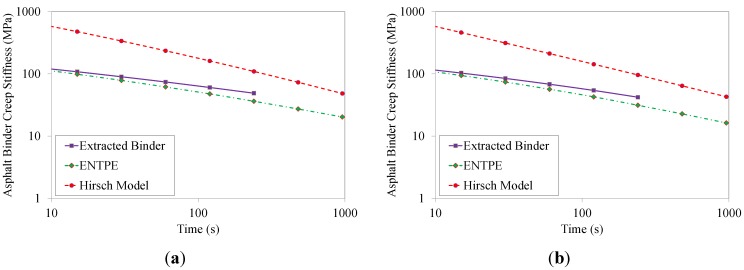
Back-calculated and extracted creep stiffness of asphalt binder: mix 7 (**a**) and 8 (**b**), T = −6 °C.

The values of the asphalt binder creep stiffness predicted from Hirsch model and ENTPE transformation were compared to the creep stiffness values obtained experimentally on the RTFOT aged original binder and on the extracted asphalt binders (mixtures 2–8). As described in Section 4, asphalt mixtures were tested for 1000 s while the test duration of asphalt binders was limited to 240 s as prescribed in the standard [39]. Therefore, in the plots of Figure 12, Figure 13, Figure 14 and Figure 15, the curves of creep stiffness obtained from asphalt binders are shorter than those predicted through back-calculation from mixture data.

Hirsch model predictions returned much higher initial stiffness values with a significant decreasing trend compared to the experimental measurements, resulting in a significant over estimation of the creep curves obtained from the extracted binders. On the other hand, the ENTPE transformation provides a very accurate prediction of the creep stiffness of the RTFOT original binder, confirming the findings of previous studies on virgin materials [45,74,76]. A common trend is observed for stiffness curves of asphalt binders obtained from the recycled mixtures (2–8): they are initially asymptotically close to the creep stiffness curves of extracted binders, while they diverge toward lower stiffness values as time increases. This suggests that the relaxation properties obtained from ENTPE back-calculation are higher compared to the extracted binders. It can be hypothesized that one of the reasons for these observations is the presence of fine particles in the asphalt binder which could not be removed during the extraction process. However, this hypothesis would result in global higher stiffness values also for the part of the curve which is asymptotically approaching the model predictions, leading to an overall deviation of the experimental measurements from the back-calculated stiffness.

An alternative and more reasonable explanation for the difference between extracted binders and back-calculated creep stiffness curves is associated with the limited or partial blending occurring between new and recycled binder. Therefore, while the mixture creep behavior is affected by all new binders and only a part of the aged, oxidized binder, the extraction and recovery process resulted in a complete blending; this implies that the resulting blend has less relaxation capability since the properties are influenced by the entire aged binder component.

Bonaquist [77] has associated the insufficient heat transfer during mixing with partial binders blending in the mixture. An additional explanation of the limited binder blending can be provided on the basis of the work recently presented by Kriz *et al.* [27]; he identified two major processes which are involved in the binder blending: contact between the two binders, achieved mechanically, and binder blending after contact is made, achieved mainly by diffusion and strongly influenced by the total new-aged binder thickness. The limited or partial blending may be linked to the distribution of the binder film thickness within the asphalt mixtures microstructure and to the inability to obtain a proper binder contact when mixing. This can explain the discrepancy between the actual binder stiffness in the mixture and that obtained from extraction.

## 7. Summary and Conclusions 

In this paper, the effect of adding different amounts of reclaimed asphalt pavement, manufactured waste scrap shingles and tear-off scrap shingles on the microstructural distribution and on the low temperature properties of asphalt mixtures was investigated. The microstructural analysis of the mixtures was coupled with the rheological investigation of both asphalt mixtures and corresponding asphalt binders. Digital Image processing and correlation functions were first used to evaluate the influence of the recycled material on the material microstructure. Then, the creep stiffness of the asphalt binder present in the mixtures was obtained with the transformation implemented by the Ecole Nationale des Travaux Publics de l’Etat (ENTPE) (coupled with Huet model) and the semi-empirical Hirsch model, respectively. Finally, the back-calculated predictions were compared to the experimental creep stiffness values measured on the short-term aged original binder and on the binders extracted from the other recycled mixtures.

The following conclusions can be drawn:

- Limited variations were observed in the 2- and 3-point correlation functions of the asphalt mixtures; however, the auto correlation length of aggregates, mastic and air voids is significantly influenced by the recycled material used. Nevertheless, while adding reclaimed asphalt pavement increases the auto correlation length, the use of recycled asphalt shingles shorten it, most likely due to the fine particles which are included in the recycled shingles.
- Smaller auto correlation length was observed for manufactured waste scrap shingles compared to tear-off scrap shingles; this indicates that larger representative volume elements are associated with the use of aged shingles.
- Hirsch predictions provide significant overestimations of the creep stiffness of original short-term aged binder and of the seven extracted asphalt binders.
- The creep stiffness of the original binder after short term aging is matched very closely by the ENTPE transformation, while the asphalt binders stiffness curves back-calculated from recycled mixtures data do not match the creep stiffness curves of extracted binders.
- It is hypothesized that blending between new and aged and oxidized binders occurred only partially due to insufficient heat transfer and limited binder contact during mixing and/or to the distribution of the binder film thickness within asphalt mixtures. Therefore, the mixture creep properties were affected by all new binders and only a portion of the old binder. On the other hand, extraction and recovery process resulted in a complete blending which implies reduced relaxation capabilities since all aged binders contributed to the properties of the blend.
- The findings of the present research indicate that the low temperature stiffness properties of asphalt mixture are only partially influenced by the spatial distribution of its components, while the blending process of asphalt binder appears to play a fundamental role when recycled asphalt materials are added to the mixture.
